# Respiratory disease complex due to mixed viral infections in chicken in Jordan

**DOI:** 10.1016/j.psj.2024.103565

**Published:** 2024-02-16

**Authors:** Mohammad Q. Al-Natour, Mohammed A. Rohaim, Rania F. El Naggar, Mohammed A. Abdelsabour, Ahmed F. Afify, Yahia M. Madbouly, Muhammad Munir

**Affiliations:** ⁎ Division of Biomedical and Life Sciences, Faculty of Health and Medicine, Lancaster University, Lancaster LA1 4YG, United Kingdom; † Department of Veterinary Pathology & Public Health, Faculty of Veterinary Medicine, Jordan University of Science and Technology (JUST), Irbid 22110, Jordan; ‡ Department of Virology, Faculty of Veterinary Medicine, Cairo University, Giza 12211, Egypt; § Department of Virology, Faculty of Veterinary Medicine, University of Sadat City, Sadat 32897, Egypt; || Department of Poultry Viral Vaccines, Veterinary Serum and Vaccine Research Institute (VSVRI), Agriculture Research Centre (ARC), Cairo 11435, Egypt; ¶ Department of Virology, Animal Health Research Institute, Agricultural Research Center, Giza, 12619, Egypt

**Keywords:** *avian influenza viruses*, *infectious bronchitis virus*, respiratory infection, poultry health, Jordan

## Abstract

The global distribution of avian respiratory viruses highlights the need for effective surveillance programs and international collaboration to monitor viral circulation and implement timely control measures. In the current study, we aim to provide a comprehensive overview of avian respiratory viral infections in the poultry flocks in Jordan, focusing on the major viruses involved, their epidemiology, clinical manifestations, and evolution based on viroinformatics that will be helpful to improve the diagnostic methods, and control strategies including vaccines in the region. In this research, various poultry broiler groups in Jordan experiencing respiratory symptoms were tested for respiratory viral pathogens from January 2021 to February 2022. The mortality rates observed in the examined groups varied between 6% and 40%. The identified strains were authenticated using the RT-qPCR assay. Furthermore, they underwent in-depth characterisation through the sequencing of the complete spike (**S1**) gene for *infectious bronchitis virus (****IBV****)* and the haemagglutinin (**HA**) gene for *avian influenza virus (****AIV****) subtype H9N2*. Co-infection of *IBV and AIV H9N2* viruses was detected through molecular analysis. The *IBV* strains showed affiliation with the variant groups GI-16 (3 strains) and GI-23 (9 strains) and exhibited numerous mutations. Meanwhile, *H9N2 avian influenza viruses* displayed various changes in amino acids within the HA gene, suggesting the influence of antibody-driven selection pressure. The phylogenetic analysis revealed that the *H9N2 viruses* identified in this investigation shared close genetic ties with EG3 (3 strains) and the Middle East group (**ME1**; 8 strains). These strains have been recently found in Jordan and nearby countries in the Middle East. Moreover, their HA genes exhibited similarities to viruses belonging to the G1-like lineage. In conclusion, avian respiratory viral infections remain a significant concern for the poultry industry, requiring constant vigilance and proactive measures to minimise their impact. Continued surveillance, robust diagnostic methods, effective vaccines, and international cooperation are essential components of a comprehensive approach to combat avian respiratory viral infections *(AI, IBV, ND and ILT ‘viruses)* and safeguard avian health and global poultry production.

## INTRODUCTION

Avian viral respiratory mixed infections refer to the simultaneous presence of multiple viral pathogens causing respiratory infections in birds. These infections can involve various avian viruses, including *avian influenza viruses (****AIVs****), Newcastle disease virus (****NDV****), infectious bronchitis virus (****IBV****), infectious laryngotracheitis virus (****ILTV****)* amongst others (Hassan et al., 2016; Yehia et al., 2023). When multiple viruses infect the respiratory system of birds, they can interact and potentially exacerbate the severity of the respiratory disease. *Avian influenza viruses* (***AIVs***) raise significant concerns owing to their capacity to infect various avian species, spanning from poultry to wild birds, and occasionally crossing over to infect humans (Rohaim et al., 2021). Coinfections involving *AIV* and other respiratory viruses can complicate the clinical presentation, increase viral shedding, and contribute to higher mortality rates in affected bird populations. The consequences of avian viral respiratory mixed infections can vary depending on the specific viruses involved, their virulence, the bird species affected, and the overall health status of the birds. In some cases, mixed infections can lead to synergistic effects, where the presence of one virus can enhance the replication or pathogenicity of another virus, resulting in more severe respiratory disease (Hassan et al*.*, 2016; Yehia et al., 2023).

Lately, there has been an increase in respiratory disease outbreaks affecting commercial chicken populations worldwide, including Jordan. These outbreaks have exhibited different mortality rates and a range of clinical manifestations. The poultry industry faces significant challenges due to the multifaceted nature of respiratory diseases (Roussan et al., 2008; Roussan et al., 2015). The poultry industry in Jordan faces challenges in implementing effective biosecurity measures due to limited awareness and resources. Inconsistent vaccination practices across farms and variations in vaccine quality contribute to disease risks. Additionally, the dynamic nature of poultry diseases requires constant updates in vaccination strategies. Collaborative efforts involving farmers, government agencies, and the veterinary sector are essential to address these challenges and ensure the industry's sustainability. Ensuring the availability of high-quality vaccines is crucial for effective disease prevention. Issues such as improper storage, handling, or administration of vaccines may compromise their efficacy, leading to incomplete protection against diseases

Chicken flocks in Jordan have been experiencing high mortality rates associated with various pathogens including *AIV*, virulent (**v**) *NDV*, and *IBV* (Fayyaz et al., 2022; Hussian et al., 2022; Rohaim et al., 2022). These pathogens hold a substantial significance and wield a notable economic influence, capable of causing disease either individually or in conjunction with one another (Roussan et al., 2008; Roussan et al., 2015). The challenges in managing poultry diseases in Jordan stem from insufficient biosecurity measures, limited vaccination coverage, and the mismatch between the strains in circulation and the vaccines currently available. Comprehensive surveillance and genetic examinations have revealed that *avian influenza subtype H9N2 viruses* have consistently maintained a presence within the poultry flocks in Jordan, which is thought to be notably impacted by asymptomatic infections in freely roaming ducks, geese, and backyard holdings that accommodate various species (Monne et al., 2007).

Between 1998 and 2010, *low pathogenic avian influenza virus (****LPAIV****) subtype H9N2*, initially isolated from Central Asia and the Middle East, could be genetically classified into 4 separate groups: A, B, C, and D. Groups A and B were found in both Central Asia and the Middle East, while groups C and D were specifically observed in the United Arab Emirates (2000–2002) and Iran (1998 - 2007), respectively (Fusaro et al., 2011). This particular subtype has been identified in mammalian hosts and has undergone evolution through a blend of point mutations and reassortment incidents, which involved *H9N2 viruses* and various other subtypes of influenza viruses (Wan et al., 2008; Chaudhry et al., 2015; Peacock et al., 2019). Consequently, it is essential to maintain ongoing surveillance and conduct thorough characterisation of these viruses to better comprehend any prevailing public health threats in this area. Recent studies have focused on exploring the evolutionary background and current situation of the G1-lineage *H9N2 subtype*. Nevertheless, there is a scarcity of information and genetic sequence data regarding *H9N2 LPAIV* specifically from Jordan. The initial appearance of the *H9N2 virus* dates back to 2003, when it was detected in both chickens and domestic ducks (Al-Natour and Abo-Shehada, 2005; Monne et al., 2007). Further examinations have revealed a significant presence of the *H9 subtype* in both broiler and layer flocks, indicating the entrenched existence of this subtype within the country (Roussan et al., 2009; Roussan et al., 2015). The identification of *avian influenza virus (****AIV****) H9N2 subtype* in chickens posed an added difficulty for the poultry sector (Roussan et al., 2009). The increasing prevalence of *AI-H9N2 viruses* among Jordan's poultry is a matter of increasing worry. Intensive surveillance indicates their continual existence, prompting inquiries into the potential consequences for avian influenza (Roussan et al., 2009; Roussan et al., 2015). The varied poultry environment, encompassing freely moving ducks and backyard facilities, serves as a source for symptomless infections, impacting avian influenza epidemiology. Since *IBV* and *H9N2 AIV* viruses are endemic in the region, it is necessary to genetically characterise these viruses to monitor the dynamic evolution and potential reassortment/recombination events. Simultaneous secondary infections involving *E. coli*, avian mycoplasma species, and nephropathogenic strains of *IBV* may expedite the infection process, leading to air-sacculitis and interstitial nephritis (Parkhe and Verma, 2021).

Despite consistent vaccination efforts employing Massachusetts (**Mass**) strains, *infectious bronchitis virus (****IBV****)* continues to exert varied effects on the poultry industry worldwide. Vaccination programs often target specific viral strains or serotypes, and the continuous monitoring of viral evolution is essential to ensure the selection of appropriate vaccine strains (Rohaim et al., 2019a; Rohaim et al., 2019b). Despite the implementation of preventive measures, challenges persist in the control of avian respiratory viral infections particularly *IBV* and *H9N2 AIV*. Factors such as viral antigenic drift, inadequate vaccine coverage, and the presence of asymptomatic carriers contribute to the ongoing circulation and persistence of these viruses (Hui, 2006). Ongoing research efforts focus on understanding viral pathogenesis, host immune responses, and the development of novel control strategies to combat these infections effectively. In addition, exploring the knowledge on viral evolution in poultry will highlight future emergence potentials and will guide the establishment of control strategies including innovative vaccines and novel antivirals. In this study, we aim to assess the current prevalence of avian respiratory pathogens, particularly *IBV* and *AIV*. Furthermore, selected respiratory viruses were genetically identified to monitor their circulating strains.

## MATERIALS AND METHODS

### Ethical Statement

The handling of samples and the steps for virus isolation strictly adhered to the standards and regulations concerning animal welfare and health, as sanctioned by the Department of Veterinary Pathology and Public Health, Faculty of Veterinary Medicine, Jordan University of Science and Technology (**JUST**), Jordan (JUST research ID: 258-2021).

### Field Samples

**Figure 1:**
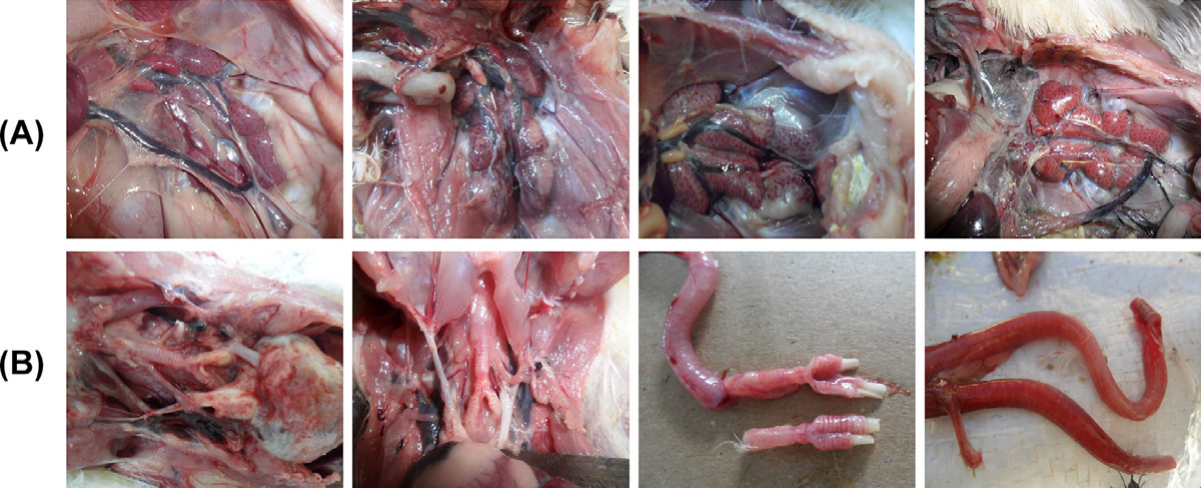
The observed postmortem findings in the chickens were as follows: (A) The kidneys displayed signs of congestion, swelling, and distinctive lobes during necropsy examination, and (B) The trachea exhibited congestion, hemorrhaging of the tracheal mucosa, and the presence of a caseous plug within the tracheal lumen.

Swabs from the trachea and/or oropharynx were taken from 86 broiler flocks that exhibited respiratory and kidney lesions including tracheitis, tracheal caseation, caseous plug and nephritis (Figure 1) between January 2021 and February 2022 (Table 1). All samples were obtained from flocks that had received vaccinations (Table 2). The study encompassed an examination across 6 provinces in Jordan; Jarash, Amman, Ajloun, Balqa, Zarqa, and Madaba. Samples collection occurred throughout different seasons, with the highest sample numbers originating from Jarash and Amman, followed by Ajloun and Balqa, and subsequently Zarga and Madaba. The distribution of samples aligned with the density of poultry farms in each respective region. A set of 10 samples per flock was gathered, the count varying across different seasons. The collection of individual swabs was performed using viral transport medium containing antibiotics (isotonic PBS, 2,000 U/mL penicillin, 2 mg/mL streptomycin, 50 μg/mL gentamycin, 50 U/mL nystatin, and 0.5% BSA). After collection, swab samples were subjected to centrifugation at 1700 rpm and 10°C for 5 min to clarify them according to guidelines provided by the OIE (Stear, 2005). The resulting supernatants were then stored at −80°C until they were utilised for subsequent analysis.

**Table 1:** Clinical history, mortalities, lesions and respiratory viruses detected in the examined chicken flocks with respiratory clinical signs in Jordan.

No.	Governorate	Mortality %	Age (days)	PM lesions	*IBV*		*H9N2*	
					RT-qPCR	Accession number	RT-qPCR	Accession number
1	Ajloun	9%	31	Tracheal caseation	+	OR515480	-	-
2	Jarash	19%	32	Tracheal caseation and nephritis	+	OR515481	+	OR504555
3	Balqa	%11	25	Tracheitis	-	-	+	OR504474
4	Amman	%15	28	Caseous Plug	+	OR515482	-	-
5	Amman	%18	25	Caseous Plug	+	OR515483	-	-
6	Jarash	%40	26	Caseous Plug and nephritis	+	OR515485	+	OR504764
7	Zaraqa	%31	26	Tracheitis, Caseous Plug	+	OR515486	+	OR505002
8	Ajloun	9%	31	Tracheitis	-	-	+	OR504503
9	Zarqa	15%	33	Tracheitis, Caseous Plug	+	OR515484	-	-
10	Amman	22%	25	Caseous Plug	+	OR515487	-	-
11	Zarqa	11%	27	Tracheitis	-	-	+	OR504504
12	Madaba	19%	35	Caseous Plug	+	OR515488	-	-
13	Madaba	21%	22	Tracheal caseation and nephritis	+	OR515489	-	-
14	Amman	8%	31	Tracheitis	-	-	+	OR504507
15	Balqa	17%	28	Caseous Plug and nephritis	+	OR515490	-	-
16	Jarash	22%	27	Caseous Plug and nephritis	+	OR515491	-	-
17	Amman	14%	30	Tracheitis	-	-	+	OR504508
18	Ajloun	12%	23	Tracheitis	-	-	+	OR504530
19	Jarash	6%	28	Tracheitis	-	-	+	OR504531
20	Balqa	7%	23	Tracheitis	-	-	+	OR504532
Total					12		11

**Table 2:** Vaccination regime used to vaccinate the Jordanian broiler flocks.

Age	Vaccination method	Used Vaccine
0	In ovo	VAXXITEK® *HVT + IBD*
1	Coarse spray	Live *NDV* (Avinew®) and live attenuated *IBV* (Poulvac IB Primer®
14	Coarse Spray	Live attenuated *IBV* (IBird®)+ Live attenuated NDV (Clone 30)
21	IM	Inactivated *NDV+H9N2* (Jova Zeit 1,7®)

### Viral RNA Extraction, Conventional RT-PCR and Sequencing

Viral RNA extraction was conducted using the QIAamp Viral RNA Mini Kit (Qiagen, Hilden, Germany) in accordance with the manufacturer's guidelines. Subsequently, single-step RT-qPCR assays for each target virus were executed using the Verso 1-Step RT-qPCR Kit Plus ROX Vial (Thermo Scientific, England, UK) along with specific oligonucleotide primers and probes. The overall reaction volume was 25 μL, comprising 5 μL of RNA template, 12.5 μL of 2 × 1-step PCR ready mix, 1.25 μL of RT-enhancer, 0.25 μL of Verso enzyme mix, 1 μL each of forward and reverse primers, 3.75 μL of nuclease-free water, and 0.25 μL of the probe for *AIVH5N1* (Slomka et al., 2007), *AIV H9N2* (Shabat et al., 2010), *IBV* (Callison et al., 2006), and *NDV* (Wise et al., 2004). The positive samples out of the RT-qPCR were further characterised and identified based on RT-PCR as previously described for *AIV H9N2* (HA gene) (Hoffmann et al., 2001) and *IBV* (S gene) (Stear, 2005; Dolz et al., 2006; Lisowska et al., 2017)**.** The decision to sequence the hemagglutinin (**HA**) gene of *H9N2* and the Spike (**S**) gene of *IBV* is strategically grounded in their pivotal roles in influenza and infectious bronchitis research, respectively. Sequencing the HA gene is essential for *H9N2* due to its involvement in host cell entry, antigenic variation, and zoonotic potential, providing insights into the virus's ability to evade the host immune response (Peacock et al., 2019). Additionally, knowledge of HA gene sequences is crucial for vaccine development, aiding in the selection of strains that closely match circulating ones to enhance vaccine effectiveness (Peacock et al., 2019). On the other hand, sequencing the S gene of *IBV* is imperative to understand how *IBV* interacts with host cells, influences disease pathogenesis, and tracks genetic diversity, particularly in hypervariable regions. This genetic information on the S gene is crucial for selecting strains for vaccine development, ensuring vaccines target prevalent genotypes for enhanced efficacy (Rohaim et al., 2019a; Rohaim et al., 2019b).

The RT-PCR assays were performed using the One Step RT-PCR Kit (Qiagen) following the manufacturer's instructions, with 5 μL of the extracted viral RNA. Each reaction consisted of 0.2 μM of each primer, 0.5 mM of the deoxyribonucleotide triphosphate mixture, and 1 μL of the one-step enzyme mixture in a final volume of 25 μL. Specific fragments were amplified with the following conditions: 30 min at 50°C; 10 min at 95°C; 40 cycles of 30 s at 95°C, 45 s at 55°C, and 1 min at 72°C; and 1 cycle of 10 min at 72°C. Subsequently, the amplification products underwent electrophoresis. Gel Red (Biotium, CA) was used to stain the gels, and the amplicons were visualised using an ultraviolet transilluminator (UVP BioImaging Systems, Cambridge, UK). The amplified RT-PCR products were purified using the PCR purification Kit (Thermo Scientific), following the instructions provided by the manufacturer. Post purification, the purified products were directly utilised for sequencing using the ABI Prism 3100 automated sequencing machine (Applied Biosystems, Foster City, CA).

Following this, every acquired sequence underwent a BLAST search using the website: https://blast.ncbi.nlm.nih.gov/Blast.cgi.

### Viroinformatics and Evolutionary Analyses

#### Mutations Mapping at S1 and HA Proteins and Functional Regions

Following, the S1 and HA nucleotide sequences underwent conversion into their respective amino acid sequences through the MEGA X program v10.1.8. This transformation was intended to enable a comparison of our reported strains at the amino acid level. To depict patterns within the aligned sequences of the hypervariable regions (HVRs), we utilised the WebLogo service (http://weblogo.threeplusone.com/create.cgi, accessed on 15 August 2023) to generate sequence logos. Sequence logos offer a more comprehensive and precise representation of S1 protein sequence similarity compared to consensus sequences. They have the ability to swiftly reveal significant features of the alignment that might otherwise be challenging to detect.

### Phylogenetic Analyses

Phylogenetic investigations were executed to probe the genetic relationships, exhibiting a pronounced clustering pattern. This pattern was dependent on the complete S1 gene of *IBV* and the HA gene of *AIV subtype H9N2*. This examination encompassed both previously documented strains and recently identified strains originating from the Middle East, including regions like Jordan and Israel, as well as other global locations. The nucleotide sequences acquired were subjected to alignments utilising the Multiple Alignment using Fast Fourier Transform (**MAFFT**) technique within Geneious software, version 11.1.3 (Biomatters, Auckland, New Zealand). Following alignment, the sequences were exported to MEGA software, version 7.0.26 (Tamura et al., 2013). Using the most suitable models for nucleotide substitution—determined by the Bayesian information criterion (BIC) to identify the models with the lowest scores—an analysis was conducted. This involved the general time reversible (GTR) model along with a discrete gamma distribution (+G) incorporating 5 rate categories and considering a fraction of sites as evolutionarily invariable (G + I). Subsequently, maximum likelihood (**ML**) phylogenetic analyses were carried out, backed by bootstrap analyses comprising 1000 replicates. The resulting trees were ultimately presented and annotated through the utilisation of FigTree software, version 1.4.2, accessible at http://tree.bio.ed.ac.uk/software/figtree/.

### S1 and HA Proteins Selective Pressure

To pinpoint conserved areas, homology models were crafted for the translated S1 and HA proteins by aligning sequences using ClustalW, integrated into BioEdit software version 7.0, through a multiple sequence alignment (**MSA**) process (Hall, 1999). The collective sections of each protein from both the field and reference strains were used in a Protein Data Bank (**PDB**) search to identify existing homologs or orthologs. The Synonymous-Non-Synonymous Analysis Program (SNAP) was utilised to gauge gene-specific dN/dS ratios for the S1 and HA genes (Kryazhimskiy and Plotkin, 2008). The tally of possible synonymous and non-synonymous alterations, alongside the actual changes in codons within each pair, was tabulated. Following this, the dN/dS ratio was calculated, comparing the rate of observed non-synonymous substitutions to that of observed synonymous substitutions. The calculations were modified to accommodate multiple hits by applying the Jukes–Cantor correction (Kryazhimskiy and Plotkin, 2008). A score exceeding 0 suggests predominant diversifying positive selection, whereas a score below 0 indicates negative selection.

## RESULTS

### *IBV* and *H9N2 AIV* Epidemiology

We conducted genetic analyses tracking the evolution of *IBV* and *H9N2 AIV* in Jordan from January 2021 to February 2022. A total of 86 samples underwent individual screening using RT-qPCR, followed by full-length amplification for S1 or HA genes for the positive samples. Among all the samples tested (Table 1), 12 out of 86 samples (13.95%) were positive for *IBV*, and 11 samples (12.79%) tested positive for *H9N2 AIV*. However, only 3 samples (3/86; 3.48%) showed mixed infections involving both *IBV* and *H9N2 AIV*. All samples tested negative for other avian respiratory viruses, including *avian avulaviruses*.

### Sequences Homology and Mutations Trend Analyses

**Figure 2:**
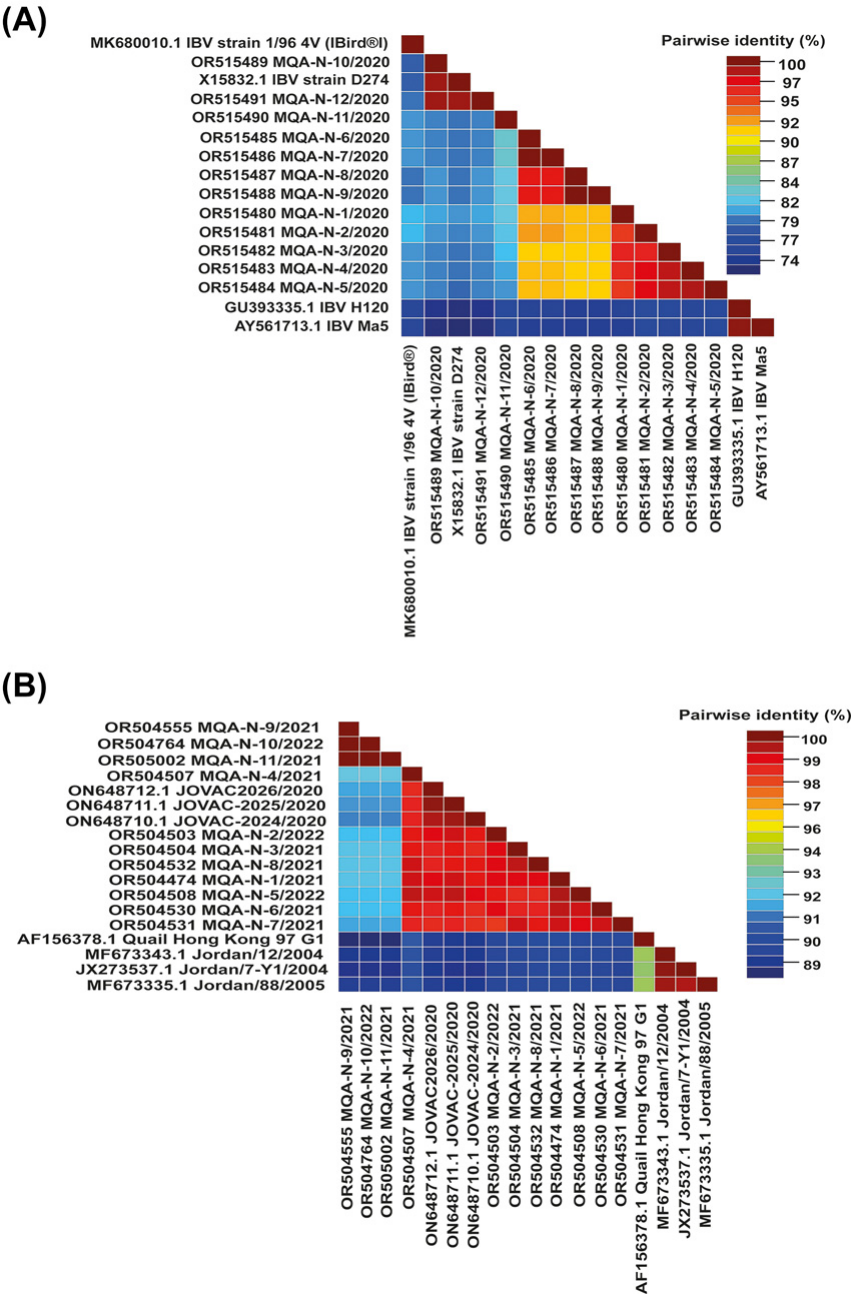
The pairwise identity plot of the spike (**S1**) (A) and haemagglutinin (**HA**) (B) glycoprotein sequences, concerning *IBV* and *H9N2 AIV*, respectively, were aligned using ClustalW and visualised through the Sequence Demarcation Tool (**SDT**) software.

The S and HA proteins in *IBV* and *H9N2 AIV* are pivotal in triggering the host's immune response and play a crucial role in generating neutralizing antibodies postvaccination. Antibodies directed against these proteins have demonstrated their vital role in neutralizing the infectivity of both *IBV* and *H9N2 AIV*. When compared, all the *IBV* strains displayed 73% and 83% amino acid identity with the *H120* or *Ma5* and *D274 IBV* vaccines, respectively **(**Figure 2A**)** that are routinely used in the country. The comparative analysis of amino acid sequences from HA genes indicated that the recent Jordanian *H9N2* strains (ON648712.1 A/chicken/Jordan/JOVAC2026/2020) were closely associated with other *H9N2* strains in the Middle East, sharing a common ancestor (AF156378.1 A Quail Hong Kong 97- G1 Lineage). Nucleotide sequence identity among the studied *H9N2 AIV* strains ranged from 84 to 98% (Figure 2B), displaying diverse levels of genetic variance compared to other representative strains of the group B- G1 lineage.

**Figure 3:**
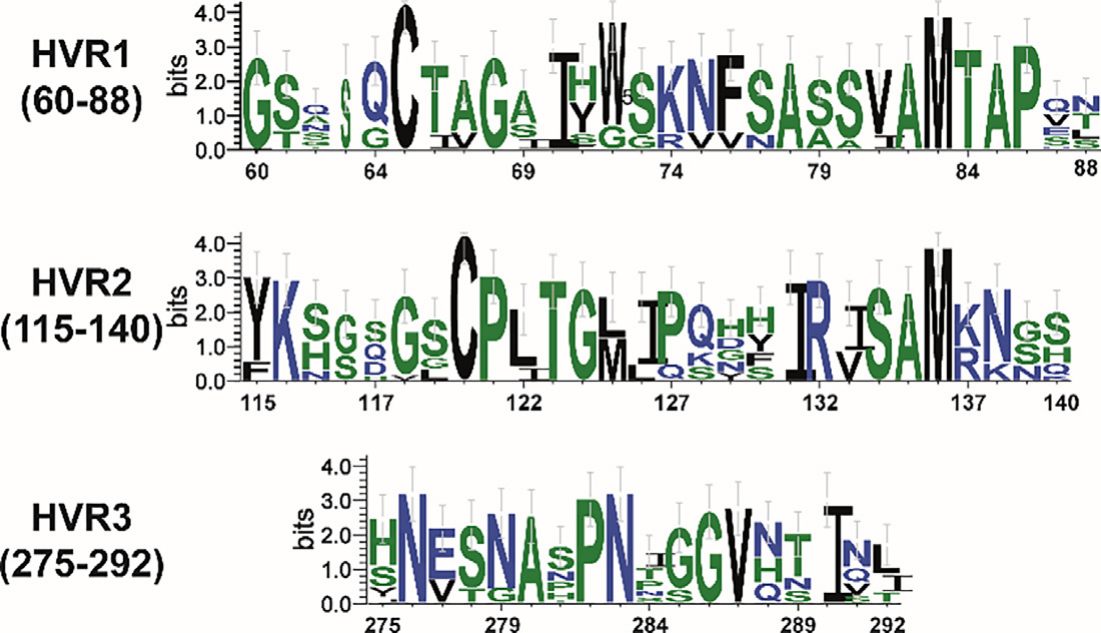
WebLogo graphics were employed to visually depict the divergence in amino acid sequences between the *IBV* strains identified in this investigation, highlighting their differences in comparison to commercially utilised vaccines (H120/*Ma5* and *D274*) as well as previously documented isolates in Jordan.

The S1 protein harboured regions of high variability termed hypervariable regions (HVRs), which were linked to the specificity of serotypes and the presence of virus-neutralizing epitopes. These HVRs were situated within specific amino acid ranges, namely HVR1 (38–67), HVR2 (91–141), and HVR3 (274–387) (Moore et al., 1997; Wang and Huang, 2000)**.** All strains of *IBV* investigated in this study differed from the other *IBV* vaccine strains utilised in Jordan. (Figure 3). It is widely recognised that even minor alterations in the amino acid sequence of the spike protein can give rise to the emergence of novel genotypes or serotypes that possess distinct antigenic properties from those of established classic and variant vaccine strains (Rohaim et al., 2019a; Rohaim et al., 2019b). Amino acid changes in the S1 protein of *IBV* can impact vaccine efficacy by introducing antigenic variations, reducing cross-protection against evolving strains, and potentially leading to vaccine breakthroughs. These changes may necessitate the development of strain-specific vaccines and continuous monitoring to keep vaccines aligned with circulating *IBV* diversity. Additionally, alterations in the S1 protein can influence virus entry, affecting pathogenicity and transmission dynamics (Stevenson-Leggett et al., 2021). Regular surveillance and adaptation of vaccination strategies are essential to address these challenges and ensure sustained effectiveness against *IBV*.

All the *H9N2 AIV* strains examined in this study showcased a cleavage site (HA0) in the HA protein with avirulent-type sequence RXXR residues, which signifies the presence of *low pathogenic H9N2 AIV* strains. Analysis predicting residues in the HA protein highlighted a typical proteolytic cleavage motif, ^335^R-S-S-R-G-L^340^, a trait characteristic of *low pathogenic avian influenza viruses (LPAIVs)*. Glycosylation motifs in *H9N2 LPAIVs* play a crucial role in antigenic variation, shielding viral proteins like hemagglutinin (**HA**) from immune recognition and aiding the virus in evading the host's immune response (Zhu et al., 2022). These motifs contribute to immune evasion by preventing antibodies from effectively binding to critical epitopes on the viral surface (Zhu et al.*,* 2022). Moreover, glycosylation patterns can influence host adaptation, impacting the virus's ability to infect different hosts and tissues (Zhu et al.*,* 2022). Changes in glycosylation motifs also affect viral fitness, influencing replication efficiency and transmission dynamics. Understanding these roles is vital for predicting outbreaks, monitoring viral evolution, and developing targeted preventive measures, including vaccines. Glycosylation motifs Asn-X-Ser/Thr (N-X-S/T, where X represents any residue except proline [**P**] and aspartic acid [**D**]) were identified within the studied strains. Specifically, these sites—29N-S-T31, 82N-P-S/T84, 105N-G-T107, 141N-V-T143, 298N-S-T300, 305N-I-T307, 492N-G-T492, and 501N-G-S503—play crucial roles in receptor binding and serve as key residues within the hydrophobic core of the stalk. Computational analyses revealed an amino acid substitution within the receptor binding site (**RBS**) and receptor pocket of the HA protein within our reported *H9N2 AIV* strains in this investigation (Table 3).

**Table 3:** Amino acid sequences analysis of the HA protein of Jordanian *H9N2 AIV* isolates.

Avian influenza viruses	Receptor binding Site (RBS)									Amino acid residues at receptor pocket (H9 numbering)	
	166	191	197	198	232	234	235	236	239	Left edge (232-237)	Right edge (146-150)
A/Quail/Hong_Kong/G1/97	S	H	T	E	N	L	Q	G	D	NDLQGR	GISRA
A/chicken/Jordan/MQA-N-1/2021	N	-	-	A	-	-	I	-	N	- G - I - -	-T- KS
A/chicken/Jordan/MQA-N-2/2022	N	-	-	A	-	-	I	-	N	- G - I - -	-T- KS
A/chicken/Jordan/MQA-N-3/2021	N	-	-	V	-	-	I	-	N	- G - I - -	-T- KS
A/chicken/Jordan/MQA-N-4/2021	N	-	-	A	-	-	I	-	N	- G - I - -	-T- KS
A/chicken/Jordan/MQA-N-5/2022	N	-	-	A	-	-	I	-	N	- G - I - -	-T- KS
A/chicken/Jordan/MQA-N-6/2021	N	-	-	A	-	-	I	-	N	- G - I - -	-T- KS
A/chicken/Jordan/MQA-N-7/2021	N	-	-	V	-	-	I	-	N	- G - I - -	-T- KS
A/chicken/Jordan/MQA-N-8/2021	N	-	-	A	-	-	I	-	N	- G - I - -	-T- KS
A/chicken/Jordan/MQA-N-9/2021	N	-	-	A	-	-	I	-	N	- G - I - -	-T- KS
A/chicken/Jordan/MQA-N-10/2022	N	-	-	A	-	-	I	-	N	- G - I - -	-T- KS
A/chicken/Jordan/MQA-N-11/2021	N	-	-	A	-	-	I	-	N	- G - I - -	-T- KS

### Immune Pressure

**Figure 4:**
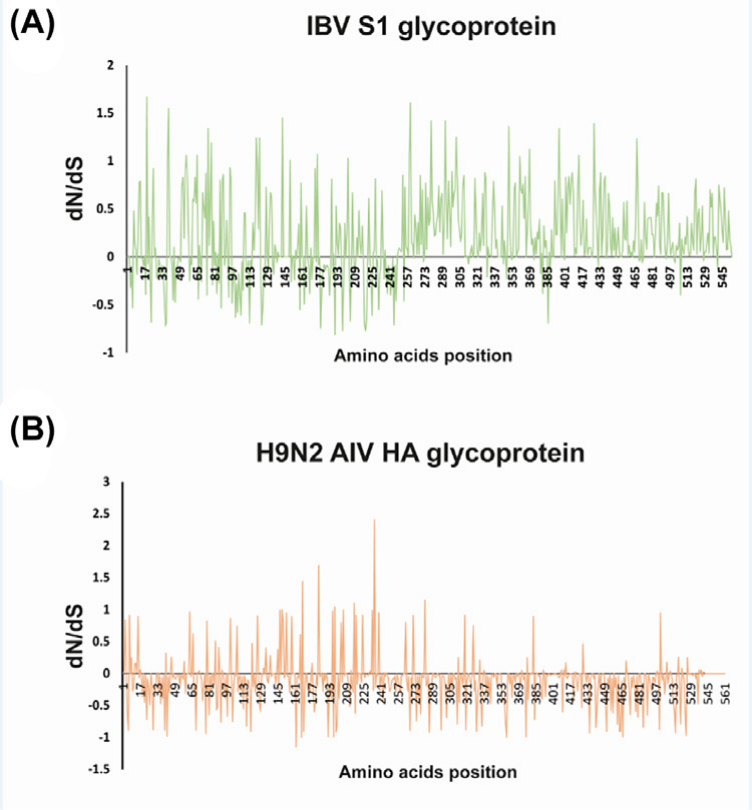
The collective dN/dS ratio was analysed, scrutinizing the average synonymous and non-synonymous substitutions, systematically examining codon by codon across the S1 (A) and HA (B) glycoproteins of *IBV* and *H9N2 AIV* strains in Jordan. This assessment incorporated the strains reported in this study, pinpointing the domains within the S1 and HA proteins most impacted by heightened selective pressure.

Mass vaccination plays a role in the collective effect that influences virus evolution, stemming from immune pressure. Our results indicated that the overall difference between the pace of nonsynonymous substitutions (**dN**) and synonymous substitutions (**dS**) for the Jordanian *IBV* and *H9N2 AIV* strains highlighted a leaning toward positive selection, particularly at pivotal positions within the S1 HVRs (Figure 4A) and HA proteins (Figure 4B), respectively. Positive selection influences the evolution of *IBV* and *H9N2 AIV*, leading to changes in viral proteins. In *IBV*, this can affect the vaccine efficacy by altering antigenic properties, requiring updates in vaccination strategies; however, positive selection in *H9N2 AIV* proteins like hemagglutinin can contributes to host adaptation and immune evasion, posing challenges for surveillance and control efforts.

### Phylogenetic Analyses

**Figure 5:**
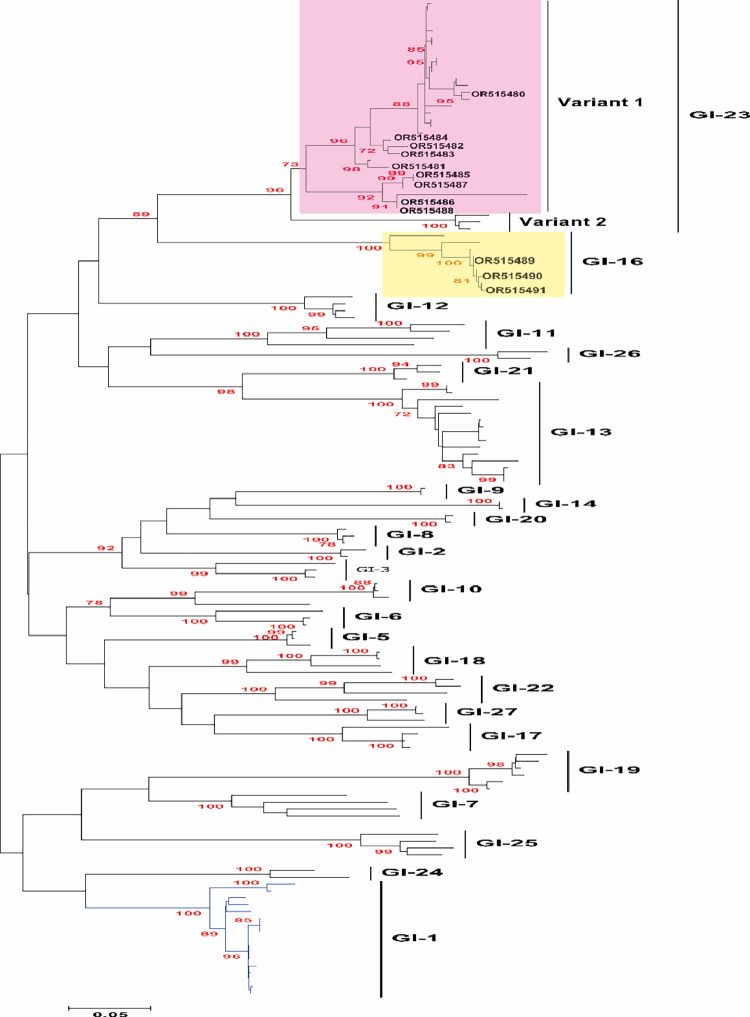
Phylogenetic investigation was conducted on the identified *IBV* strains to observe their clustering tendencies in relation to representative *IBV* lineages, genotypes, or serotypes, utilizing the complete S1 gene. The observed strains were classified into 2 distinct sublineages: nine strains were affiliated with variant 1 of the GI-23 lineage, while three strains fell within the GI-16 lineage. Unrooted phylogenetic trees were generated utilizing the distance-based maximum likelihood method and MEGA 7 software. To validate the branches within the tree, statistical analysis was performed through bootstrap analysis employing 1000 replications of bootstrap re-sampling, where numbers above branches represent neighbour-joining bootstrap values equal to or greater than 80%. The proportionate tree was depicted with branch lengths measured in substitutions per site. The *IBV* strains associated with variant 1 of the GI-23 lineage were highlighted within a light pink box, while those connected to the GI-16 lineage were enclosed in a light yellow box.

**Figure 6:**
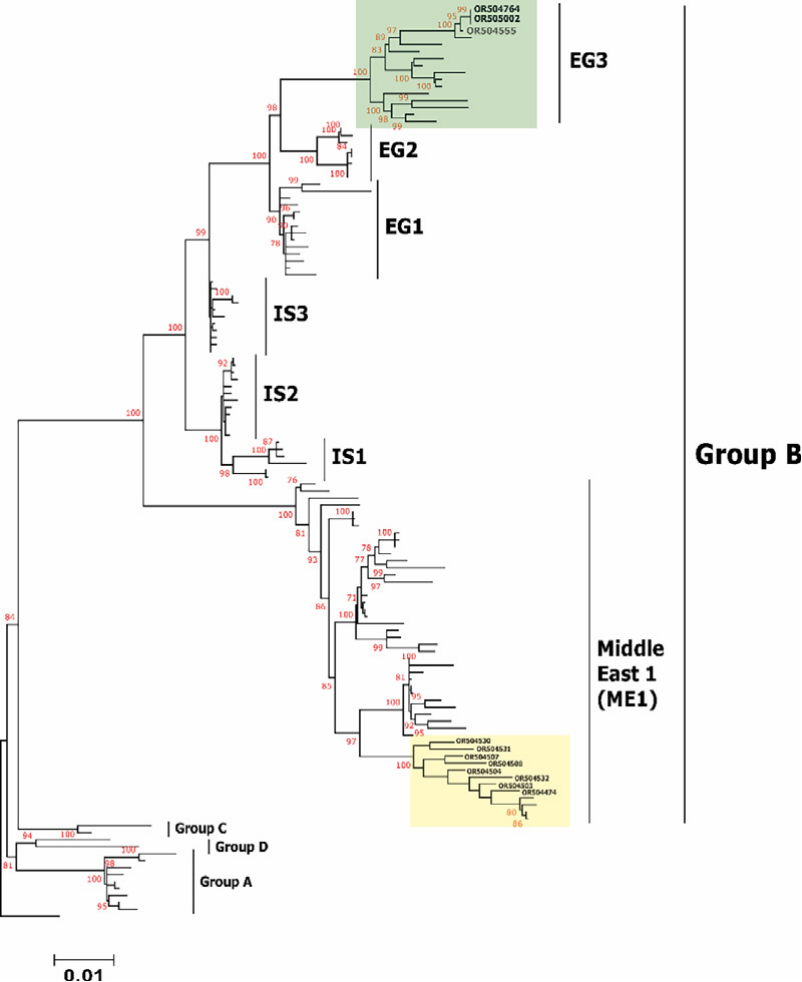
Phylogenetic examination was conducted on the studied *H9N2 AIV* strains, evaluating their clustering tendencies in relation to distinct subgroups or subclustering within the comprehensive HA gene. The identified strains were categorised into 2 separate subgroups: 3 strains were grouped within the sublineage EG3, while eight strains were clustered within the Middle East 1 (**ME1**) sublineage. The rooted phylogenetic trees were created using the distance-based maximum likelihood method and MEGA 6 software. Statistical validation for the tree branches was appraised through bootstrap analysis using 1000 replications of bootstrap resampling. The numbers displayed above branches signify neighbour-joining bootstrap values of ≥80%. The tree was drawn proportionally, depicting branch lengths measured in substitutions per site. The *H9N2 AIV* strains affiliated with the EG3 sublineage were highlighted within a light green box, whereas those linked to the ME1 sublineage were enclosed in a light-yellow box.

To analyse the epidemiological grouping of the Jordanian *IBV* and *H9N2 AIV* strains in this study, genome sequences representing these viruses were acquired from the National Center for Biotechnology Information (**NCBI**) databases. These sequences were utilised for both phylogenetic and comparative genomic investigations. A Bayesian consensus phylogenetic analysis was conducted, and the results were corroborated using the neighbour-joining method. For *IBV*, the clustering pattern was as follow; nine strains within variant 1 of GI-23 lineage and 3 strains with GI-16 lineage (Figure 5). On the other hand, *H9N2 AIV* strains reported in this study were clustered as follow; 3 strains with EG3 sub-lineage of group B and eight strains within Middle East 1 (ME1) sub-lineage of G1 lineage along with previously reported strains from Jordan, Israel, Gulf countries and some Middle East countries (Figure 6). Interestingly, the Jordanian viruses clustered together to set up a separate branch compared to other viruses of the same ME1 sub-lineage (Figure 6). Meanwhile, all the *H9N2 AIV* strains that belong with EG3 of group B reported here were mixed infected with *IBV* strains, which may indicate the correlation between this *H9N2* sublineages and the mixed infection with *IBV* and or other respiratory viruses for *IBV* and *H9N2 AIV* epidemiology and evolution.

Diverse sublineages within the *H9N2 avian influenza viruses* exhibit variations in genetic characteristics, influencing factors such as pathogenicity and host interactions. Co-infections, whether with bacteria like E. coli or other viral pathogens such as *IBV*, are common in poultry. The specific *H9N2* sublineage involved in mixed infections can impact pathogenic outcomes, and the virus's immunosuppressive effects may enhance susceptibility to secondary infections. Antigenic drift within *H9N2* sublineages may affect vaccine efficacy, especially in the context of co-infections. Understanding these correlations is crucial for disease control, vaccination strategies, and assessing potential zoonotic risks, highlighting the intricate dynamics of avian influenza.

## DISCUSSION

In the current research, the examined flocks exhibited diverse mortality rates ranging from 6 to 40%, attributable to suspected symptoms of *IBV* and or *H9N2 AIV* infection, notably presenting with tracheitis and the presence of caseous plugs. All samples tested negative for other respiratory viruses, including *NDV, ILTV,* and the *avian influenza H5N1 subtype* (Azeem et al., 2022; Naz et al., 2022). Mixed infections with avian viruses can significantly influence the evolutionary trajectory of these pathogens. Despite being known as “low pathogenic,” *avian influenza virus* (***AIV***) subtype *H9N2* triggers frequent outbreaks with noteworthy mortality rates and substantial economic implications across diverse poultry production systems worldwide. Numerous documented instances highlight this impact (Nili and Asasi, 2003; Parvin et al., 2015; Al-Garib et al., 2016). Infections involving respiratory pathogens like field *IBV* strains or live *IBV* vaccines have demonstrated the ability to exacerbate virus pathogenicity and prolong the shedding of *H9N2 virus*. This enhancement of pathogenicity is attributed to a trypsin-like serine domain encoded by *IBV*, as previously reported (Ng and Liu, 2000). Previous studies have indicated a prolonged and intensified *H9N2* viral shedding in chickens immunised with live *IBV* vaccine (Haghighat-Jahromi et al., 2008; Tavakkoli et al., 2011). Thus, the potential role of live vaccine administration in influencing pathogenesis during co-infections holds significant implications for animal health and necessitates comprehensive exploration. Live vaccines can stimulate an immune response, aiding in the control and prevention of infections. However, the intricate interactions between live vaccines and co-infecting pathogens may vary, affecting replication, virulence, and overall pathogenesis to modulate the severity and duration of infections, clinical signs, viral shedding, and tissue damage. Therefore, strategic vaccine design and administration are essential to ensure effective protection against targeted pathogens while minimizing any unintended consequences in the context of co-infections.

The serotype determinants of *IBV* have been traced within the initial 395 amino acids of the S1 subunit, housing 3 primary hypervariable regions (**HVRs**). These regions, found between amino acid residues 38 to 67 (HVR-1), 97 to 141 (HVR-2), and 274 to 387 (HVR-3), are closely linked with virus-neutralizing antigenic site (Moore et al., 1997). This study detected amino acid changes within the virus neutralization crucial antigenic epitopes in the *IBV* strains, specifically at positions 296 and 378 (PRL). These substitutions have previously been identified as causing decreased vaccine efficacy and the emergence of *IBV* escape mutants (Yu et al., 2001). Moreover, the extensive utilization of diverse vaccines, alterations in serotypes, and genetic variations can impose pressure that leads to the rise of novel genetic variations, consequently influencing alterations in the virus's tissue tropism and pathogenicity (Cavanagh et al., 1992a; Cavanagh et al., 1992b). It is important to note that relying solely on RT-qPCR assay does not provide differentiation among field strains, vaccine strains, and even within field strains themselves for *IBV*. Hence, S1 gene sequencing remains the sole method capable of distinguishing between all strains/genotypes of *IBV*. In this study, comprehensive phylogenetic analysis of the full-length S1 gene revealed that nine*IBV* strains fell within variant 1 of the GI-23 lineage, while 3 strains showed close association with the GI-16 lineage.

The present study's data indicate that even in vaccinated flocks, *IBV* alone can lead to significant mortality rates. Mortality rates were further exacerbated when mixed infection with *IBV* and *H9N2 AIV* (≥40%), even in flocks that were vaccinated against both viruses. Moreover, the *H9N2 avian influenza virus* led to notable respiratory manifestations, tracheal caseation, and a mortality range of 6 to 14%. Concurrent infections involving *LPAI-H9N2* with other respiratory pathogens (primarily *IBV*, Mycoplasma gallisepticum, and Escherichia coli) are thought to amplify the severity of clinical conditions, subsequently leading to elevated mortality rates (Nili and Asasi, 2003; Haghighat-Jahromi et al., 2008).

Since 1999, the G1-like lineage of *H9N2 avian influenza viruses*, including groups A and B, has remained widespread in the Middle East (Bashashati et al., 2013). All the Jordanian *H9N2 AI* strains identified in this research are part of the G1-like lineage's group B (Monne et al., 2013; Roussan et al., 2015). Although the documented *H9N2 viruses* exhibit minor genetic changes within their HA gene, specific amino acid alterations indicate the impact of antibody-driven selection pressure on these viruses within their native settings. Significant substitutions encompass those situated within the intersecting area of the antigenic epitope, previously documented in reports (Kaverin et al., 2004) and within receptor binding sites 128 and 180 (based on H9 numbering) (Wan et al., 2008). In 2013, a genetic variation of the *H9N2 virus*, distinct from the vaccine strain, was discovered in vaccinated broiler flocks. These flocks displayed heightened mortality rates and severe clinical symptoms (Gharaibeh and Amareen, 2015). Consequently, there was a recommendation to update the *H9N2* vaccines in Jordan to bolster the level of protection provided (Gharaibeh and Amareen, 2015). It is important to note that the specific impact of viral mixed infections on the evolution of avian viruses can vary depending on several factors, including the characteristics of the viruses involved, the host's immune response, and the ecological context in which the infections occur.

Understanding the nature of the interaction between *AIV H9N2* and *IBV*, whether cooperative or inhibitory, demands meticulous investigation. This assessment should encompass diverse infection timings, spanning both concurrent and sequential administrations to authentically replicate real-world scenarios. Furthermore, a comprehensive analysis of vaccines targeting each disease, including both live and inactivated vaccines is crucial. The timing of vaccine administration must be strategically determined, accounting for the presence of maternally derived antibodies, to ensure these antibodies do not hinder the effectiveness of the administered vaccines, ultimately minimizing clinical symptoms under field conditions. Meanwhile, understanding viral evolution and up to date monitoring data will not just help us to understand the evolution of avian viruses, but also show that such cofactors contribute to their ongoing circulation and correlation between gene cartography and antigenic virus mapping (e.g., vaccines failure, virus evolution). The viroinformatics and evolutionary approaches are used to map the key factor of avian viruses, this information would be essential for selection of seed virus for vaccine updates and vaccinations schedule update.

Understanding the impact of mixed infections on avian viruses' evolution is complex and depends on various factors like virus characteristics, host immunity, and ecological context. Investigating the interaction between *AIV H9N2* and *IBV*, whether cooperative or inhibitory, requires thorough examination with different infection timings. Comprehensive analysis of vaccines for each disease, including live and inactivated ones, is crucial, considering the strategic timing of administration to overcome maternal antibody interference. Monitoring viral evolution and utilizing viroinformatics aid in selecting effective vaccines, updating vaccination schedules, and comprehending ongoing virus circulation. Additionally, it is essential to consistently monitor and survey *H9N2 AIV* and *IBV* to understand the sequence features of dominant strains. This information is crucial for refining and enhancing vaccination strategies, aiming to develop or select vaccines that can provide cross-protection against the majority of circulating genotypes. Meanwhile, understanding *LPAI H9N2* genetic traits is crucial for effective surveillance and control.

## CONCLUSIONS

This study reveals the ongoing evolution of *H9N2* and *IBV* respiratory viruses in Jordan, including instances of co-infections. The frequent co-infection between *H9N2 LPAIV* and *IBV* underscores the need to explore potential synergistic mechanisms between these viruses. *H9N2 LPAIV* and *IBV* collectively contribute to respiratory diseases in Jordan, either independently or in co-infections, often leading to high mortality rates. The rapid evolution of *IBV* in hypervariable regions (**HVR**), especially within variant 1 of the GI-23 lineage and GI-16 lineage, has been observed. Circulating *H9N2* strains exhibit mutations in antigenic and receptor binding sites. These *H9N2* and *IB viruses* differ from vaccine strains, necessitating a comprehensive study on their genetic evolution and its impact on pathogenicity and vaccine efficacy.

Phylogenetic analysis of *H9N2 viruses* in this study reveals genetic connections with EG3 (3 strains) and the Middle East group (ME1; 8 strains), previously identified in Jordan and neighboring Middle Eastern countries. The HA genes of these viruses also display similarities to those in the G1-like lineage. Continuous monitoring of sequence variations is crucial for adapting vaccination strategies to provide cross-protection against prevailing genotypes. The implications extend beyond poultry, emphasizing the necessity for a One Health approach. Collaborative efforts between veterinary and public health authorities are essential to address potential zoonotic risks, underscoring the dynamic nature of infectious diseases.
